# 5T4 oncofoetal glycoprotein: an old target for a novel prostate cancer immunotherapy

**DOI:** 10.18632/oncotarget.17666

**Published:** 2017-05-07

**Authors:** Federica Cappuccini, Emily Pollock, Stephen Stribbling, Adrian V.S. Hill, Irina Redchenko

**Affiliations:** ^1^ The Jenner Institute, University of Oxford, Roosevelt Drive Oxford, Oxford OX3 7DQ, United Kingdom

**Keywords:** cancer, viral vectored vaccine, 5T4 oncofoetal antigen, immunogenicity, immune checkpoint blockade

## Abstract

The tumour-associated antigen 5T4 is an attractive target for cancer immunotherapy. However to date, reported 5T4-specific cellular immune responses induced by various immunisation platforms have been largely weak or non-existent. In the present study, we have evaluated a heterologous prime boost regime based on the simian adenovirus ChAdOx1 and modified vaccinia virus Ankara (MVA) expressing 5T4 for immunogenicity and tumour protective efficacy in a mouse cancer model. Vaccination-induced immune responses were strong, durable and attributable primarily to CD8+ T cells. By comparison, homologous MVA vaccination regimen did not induce detectable 5T4-specific T cell responses. ChAdOx1-MVA vaccinated mice were completely protected against subsequent B16 melanoma challenge, but in therapeutic settings this regime was only modestly effective in delaying tumour outgrowth. Concomitant delivery of the vaccine with monoclonal antibodies (mAbs) targeting immune checkpoint regulators LAG-3, PD-1 or PD-L1 demonstrated that the combination of vaccine with anti PD-1 mAb could significantly delay tumour growth and increase overall survival of tumour-bearing mice. Our findings support a translation of the combinatorial approach based on the heterologous ChAdOx1-MVA vaccination platform with immune checkpoint blockade into the clinic for the treatment of 5T4-positive tumours such as prostate, renal, colorectal, gastric, ovarian, lung cancer and mesothelioma.

## INTRODUCTION

5T4 is an oncofoetal glycoprotein that belongs to the family of shared tumour antigens. It was identified in 1990 by searching for shared surface molecules of human trophoblast and cancer cells, with the rationale that they may have a function in survival of the foetus as a semi-allograft [1]. Since then, this antigen has been a subject of intensive exploration as a potential target for cancer immunotherapy. A rationale for the development of 5T4-based vaccines is underpinned by the high expression of this antigen in a wide range of human solid malignancies [1–3] and an apparent correlation of its expression with disease progression [4, 5].

The first clinical testing of the 5T4-targeting vaccine started more than a decade ago, with the 5T4 protein expressed from the modified vaccinia Ankara virus (MVA). This vaccine was administered to late stage colorectal cancer patients as a homologous prime-boost vaccine known under the trade name of TroVax [6]. To date, TroVax has been given to over 500 patients with colorectal, breast, renal, prostate cancer and mesothelioma in the course of phase I–III clinical trials [7, 8]. Although the TroVax safety profile was good and the vaccine was well tolerated, vaccine-specific cellular immune responses and clinical efficacy were modest, with a trend toward improved progression-free survival in those patients with the highest 5T4-specific antibody titres [9, 10].

However, T cells are known to be important in immune control of cancer, and a significant body of evidence accumulated over the last two decades have shown that prime-boost protocols involving sequential administration of different vectors encoding the same antigen(s) yield considerably higher immune responses with protective capability in several animal models and clinical trials. In fact, a vaccination strategy based on the simian adenovirus prime and MVA boost (pioneered in our laboratories over a decade ago) proved to be the most powerful approach for the induction of polyfunctional protective T cell responses against some human pathogens in clinical trials [11–16].

We have previously extended this approach to test the ability of the simian adenovirus and MVA vaccination regime to break tolerance to the tumour associated self-antigen STEAP1 in a mouse model of prostate cancer. In that study, we demonstrated that protection against tumour outgrowth was mediated by the induced T cell responses to the vaccine transgene [17]. Encouraged by these data, we have been undertaking a clinical evaluation of the heterologous prime-boost regime based on this simian adenovirus, ChAdOx1, and MVA virus, both expressing 5T4. The recruitment to a phase I clinical trial in intermediate risk prostate cancer to assess the safety, immunogenicity and early clinical efficacy of this first-in-man vaccine is currently underway (NCT02390063). In order to support future combinatorial clinical trials, we have also evaluated whether a combination of the ChAdOx1-MVA 5T4 vaccination platform with immune checkpoint blockade would increase vaccine tumour protective efficacy in a mouse model. To this end, we have tested ChAdOx1-MVA 5T4 vaccine protective efficacy with and without checkpoint inhibitors (CPIs) in the B16 melanoma model.

The experimental findings presented here demonstrate that although ChAdOx1-MVA 5T4 vaccine as a monotherapy completely protects mice against subsequent tumour challenge, it is modestly effective in preventing outgrowth of established tumours. However, a combination of the vaccine with anti PD-1 antibody significantly delays tumour growth of aggressive B16 melanoma cells even in therapeutic settings and increases overall survival. In this study, we have demonstrated for the first time that ChAdOx1-MVA vaccination regime is much more effective in inducing strong 5T4-specific T cell responses compared to the previously tested vaccines targeting 5T4 [18, 19]. Our findings also provide a rationale for using CPIs in combination with antigen-specific immunotherapy for the treatment of prostate cancer, a cancer type for which CPIs as a monotherapy have not yet shown clinical efficacy [20].

## RESULTS

### Immunogenicity of viral vectors encoding h5T4 in C57BL/6 mouse strain and efficacy of the 5T4-targeting vaccine in the TRAMP-C1 tumour challenge model

**Figure 1:**
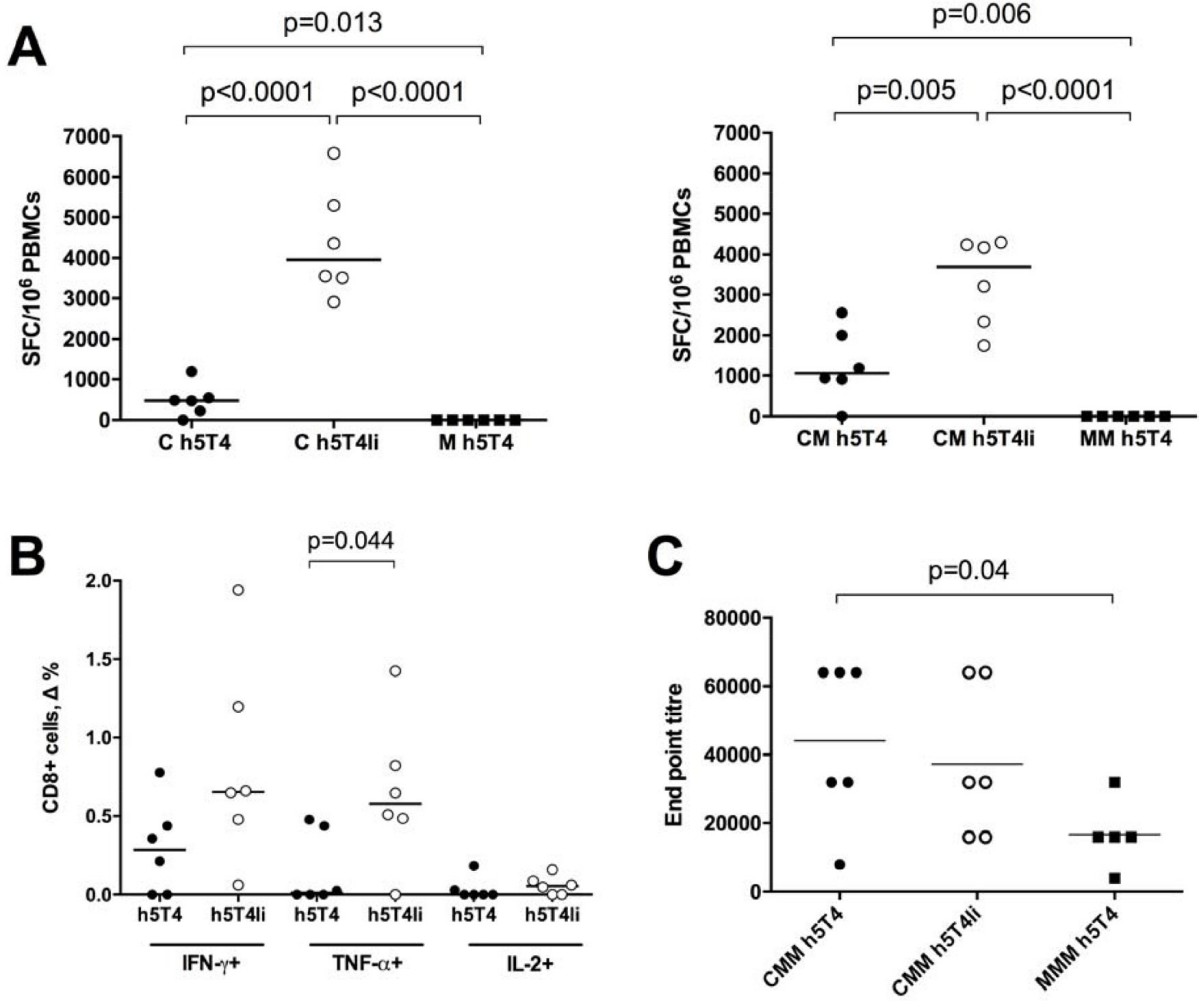
Heterologous ChAdOx1-MVA vaccination regime induces cellular and humoral immune responses against h5T4 C57BL/6 mice were immunised intramuscularly at three week intervals with 10^8^ IU of ChAdOx1 vectors expressing unmodified h5T4 antigen or h5T4 fused to the invariant chain (Ii), followed by 10^7^ pfu of MVA vectors expressing corresponding transgenes, or were given a homologous MVA.h5T4 prime-boost at 10^7^ pfu. (**A**) Graphs show representative data of *ex vivo* blood ELIspot performed after prime (left panel) and prime-boost (right panel) immunisations. Bars represent median spot forming cells (SFC) per 10^6^ PBMCs. (**B**) Intracellular cytokine staining (ICS) was performed on PBMCs isolated from mice following the heterologous prime-boost. The graph shows percentage of CD8+ T cells secreting IFN-γ, TNF-α and IL-2 in response to *in vitro* stimulation with h5T4 peptide pool. Δ values are calculated by subtracting values obtained in unstimulated cells. Bars represent median. (**C**) Anti 5T4 antibody titres were measured by end point ELISA after the third vaccination. C = ChAdOx1, M = MVA. Significant *p* values are shown. Bars represent median.

It has been shown in the past that cellular immune responses against h5T4 antigen following a homologous MVA vaccination regime have been weak [18] or non-existent [19] in mouse models. In the first experiment, we set out to investigate whether a heterologous prime-boost immunisation regime based on the ChAdOx1 and MVA recombinant viral vectors, which has previously resulted in breaking tolerance to the STEAP1 self-antigen [17], is capable of eliciting T cell responses against h5T4 in mice. Also, in order to improve immunogenicity, viral vectors were designed to express the modified antigen by direct linkage to the MHC class II-associated invariant chain (Ii), as such fusions have been demonstrated to improve protective immune responses against certain tumour antigens [21, 22]. To assess immunogenicity of the native h5T4 and h5T4Ii fusion constructs, C57BL/6 mice were prime-boosted at 3–4 week intervals with ChAdOx1-MVA or MVA-MVA expressing the respective antigens. After each vaccination, an *ex vivo* IFN-γ ELIspot assay was performed on PBMCs using a pool of h5T4 peptides covering the entire protein. As shown in Figure 1A (left panel), h5T4-specific T cell responses could be detected after the priming vaccination with ChAdOx1 vectors expressing both the unmodified h5T4 and h5T4Ii fusion. Of note, the highest frequencies of antigen-specific T cells were obtained in mice vaccinated with the Ii-fused h5T4. In contrast, a single immunisation with MVA.h5T4, or even MVA.h5T4Ii did not elicit antigen-specific IFN-γ responses (data not shown). The MVA boost further increased the magnitude of h5T4-specific responses in ChAdOx1 primed mice; however, h5T4-specific T cells in the MVA-MVA group were not detected (Figure 1A, right panel).

We have also measured immune responses following the ChAdOx1.5T4 prime and MVA.5T4 boost in the BALB/c mouse strain, and found that they were detectable only after the boosting immunization and were of a lower magnitude compared to C57BL/6 mice. We have also used the ChAdOx1-MVA vaccination regime to immunise mice against the murine 5T4 antigen in its native and Ii-fused forms, and, unsurprisingly, only sporadic responses were detected, likely because of self tolerance. On average these responses were one log lower than the responses against the human antigen (data not shown).

Specific responses to h5T4 in the ChAdOx1-MVA vaccinated groups were further analysed by flow cytometry in order to assess the relative contribution of CD4+ and CD8+ T cells. Representative results are shown in Figure 1B. After the respective boost vaccinations, PBMCs were interrogated for their expression of IFN-γ, TNF-α and IL-2. The majority of mice showed 5T4-specific secretion of these cytokines by CD8+ T cells, with a trend of increased production of IFN-γ and IL-2, and a significant increase of TNF-α secretion when mice were vaccinated with the Ii-fused antigen. On the contrary, we could detect a very low percentage of CD4+IL-2+ T cells in only one mouse from the h5T4Ii group (data not shown).

5T4-specific antibody responses after vaccination were also measured. To this end, mice were primed either with ChAdOx1 or MVA vectors and boosted with MVA twice at 3-week intervals. As shown in Figure 1C, the end point titres of 5T4-specific antibodies were significantly higher following a heterologous regime compared to homologous prime-boost vaccination.

**Figure 2:**
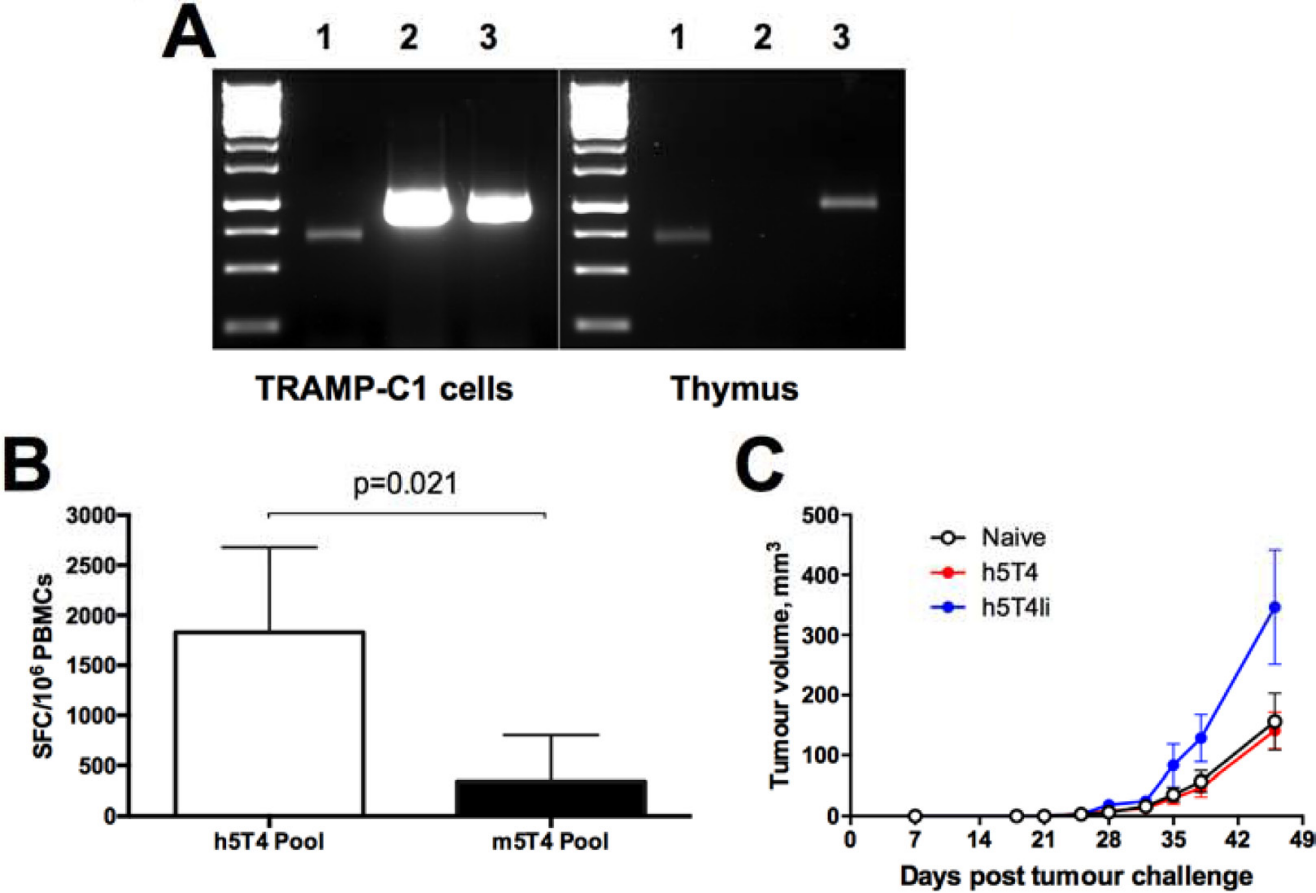
Immune responses induced against human 5T4 antigen do not protect against tumours expressing murine 5T4 despite *in vitro* cross-reactivity (**A**) Expression of murine 5T4 on transcriptional level in TRAMP-C1 cells and murine thymus by PCR. After total RNA extraction, RT-PCR with primers specific for β-actin (lane 1), STEAP1 (lane 2) and m5T4 (lane 3), was performed. (**B**) Cross-reactivity with m5T4 protein of h5T4 specific murine T cells. Blood samples were collected from mice vaccinated with 10^8^ IU of ChAdOx1.h5T4 followed by 10^7^ pfu of MVA.h5T4; PBMCs were stimulated with h5T4 or m5T4 peptide pools and tested by ELIspot assay. Bars represent mean responses + SD. (**C**) C57BL/6 male mice were injected subcutaneously with TRAMP-C1 cells and randomised into three groups. The first group received GFP vaccination (naïve) and the second and third groups received h5T4 and h5T4Ii encoding vaccines, respectively. Tumour size was measured three times per week and volumes were calculated via the equation (a^2^*b)*0.52. Kinetics of tumour growth for each group are expressed by mean tumour volume ± SEM. Data from one representative experiment are shown.

In order to investigate whether h5T4-specific T cell responses detected after the heterologous vaccination could protect against tumour outgrowth, a challenge study was carried out using the syngeneic TRAMP-C1 cell line. This prostate cancer cell line typically expresses a number of murine prostate-associated antigens, such as PAP, PSCA and STEAP1, and has been used for testing protective efficacy of cancer vaccines targeting those antigens [23, 24]; however, TRAMP-C1 cells have not been characterized in terms of 5T4 expression. To assess the utility of the TRAMP-C1 tumour cell line for testing efficacy of the 5T4-targeting cancer vaccines, we have evaluated m5T4 expression in TRAMP-C1 cells by RT-PCR. As shown in Figure 2A (left panel), the 5T4 mRNA transcript was indeed amplified from total TRAMP-C1 cell RNA, as well as one of the prostate-specific antigens, STEAP1, and the control β-actin transcript. We have also demonstrated m5T4 expression in the murine thymus as opposed to STEAP1 (Figure 2A, right panel), which may explain the poor immunogenicity of viral vectors encoding murine 5T4 antigen compared to the STEAP1-targeting vaccine in mice [17].

Because we have detected some cross-reactivity of h5T4-specific T cells with the murine antigen, in that they can recognise antigen-presenting cells loaded with the murine 5T4 peptide pool in *ex vivo* IFN-γ Elispot (Figure 2B), we next tested whether strong immunity against h5T4 antigen and T cell cross-reactivity are sufficient for controlling outgrowth of m5T4-positive tumours. To this end, mice were prime-boosted with ChAdOx1-MVA expressing h5T4 or h5T4Ii, bled to confirm the induction of 5T4-specific T cell responses (data not shown) and then inoculated subcutaneously with TRAMP-C1 cells. Control mice were left unvaccinated before tumour challenge. The tumour volume was monitored and measured at regular intervals throughout the experiment. Figure 2C shows tumour growth kinetics in the 3 groups of mice. Disappointedly, though not completely unexpectedly, TRAMP-C1 tumours in vaccinated mice progressed at the same rate as in naïve controls, or even faster in case of h5T4Ii-vaccinated mice. These data imply that the elicited h5T4-specific T cell responses are not protective against tumours expressing the murine antigen despite 83% nucleotide identity of the human and mouse 5T4 coding regions [25].

### Prophylactic efficacy of the h5T4-targeting vaccine in the B16.h5T4 tumour challenge model

**Figure 3:**
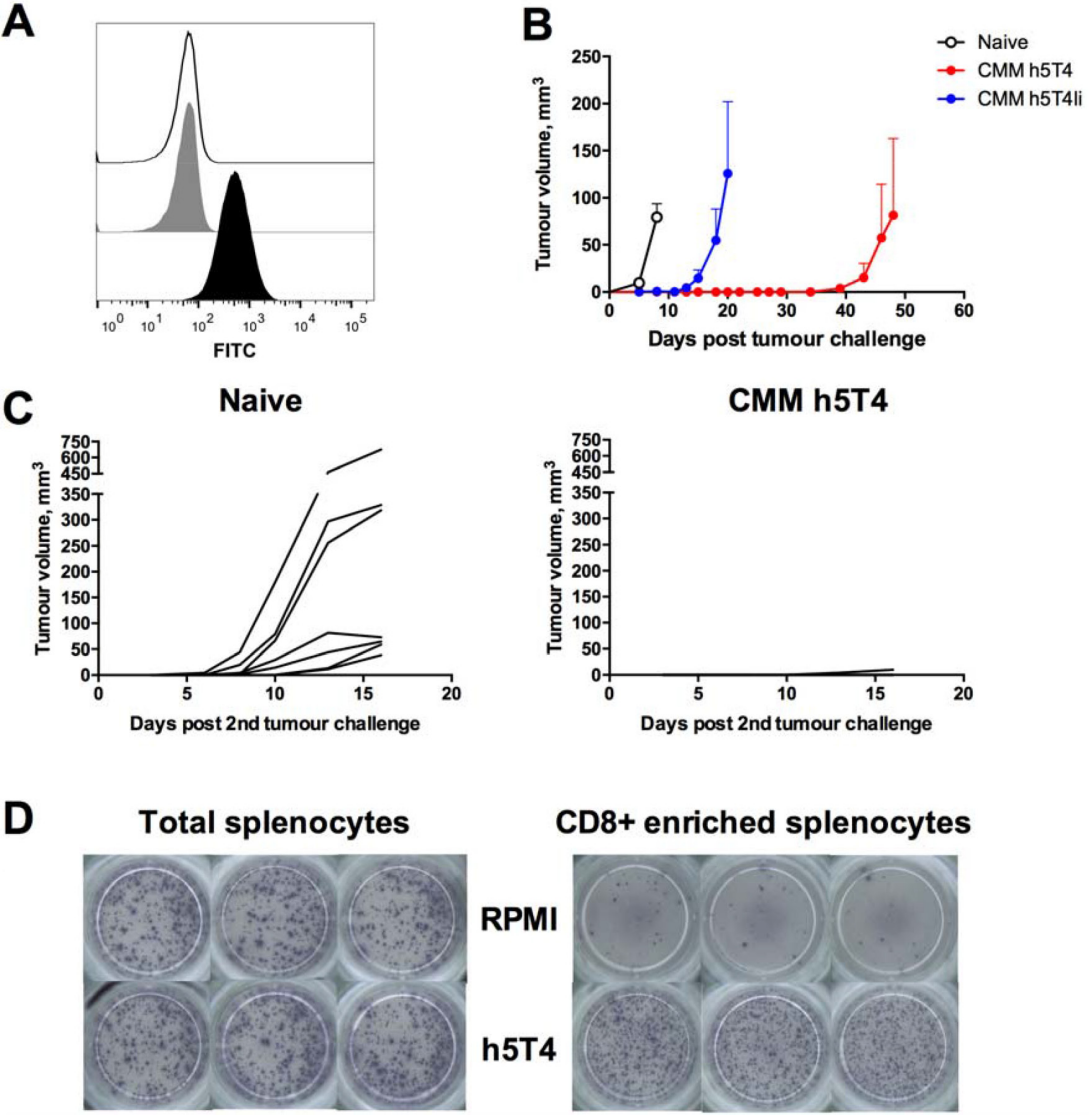
Prophylactic vaccination against h5T4 is highly protective in B16.h5T4 melanoma tumour model (**A**) Flow cytometric analysis of h5T4 expression in transfected B16 cells (black line: isotype control antibody; grey filled line: unstained; black filled line: anti h5T4 antibody). (**B**) C57BL/6 mice were immunised intramuscularly at three week intervals with 10^8^ IU of ChAdOx1 vectors followed by 10^7^ pfu of MVA vectors encoding h5T4 or h5T4Ii. Control mice were left untreated (naïve). One week after the second MVA boost, mice were challenged subcutaneously on the right flank with 0.5*10^6^ B16.h5T4 cells. Tumour size was measured three times per week and volumes were calculated as described above. Tumour growth kinetics for each group are expressed by mean tumour volume ± SEM. (**C**) Re-challenge of h5T4 vaccinated mice. 0.5*10^6^ B16.h5T4 cells were injected subcutaneously on the left flank of surviving h5T4 vaccinated mice. An additional group of naïve mice was used as control. Tumour growth kinetics for each individual mouse in the two groups are shown. Representative data of three biological replicate experiments are shown. (**D**) Representative pictures of ELISpot plate wells. At the end of the experiment, CD8 positive T cells were isolated by negative selection from splenocytes of h5T4 vaccinated mice and stimulated with the 5T4 peptide pool in an ELISpot assay.

Having found the TRAMP-C1 tumour model unsuitable for testing efficacy of the h5T4-targeting vaccines, we moved on to evaluate prophylactic efficacy of h5T4 vaccination in mice by taking advantage of a tumour cell line stably transfected to express h5T4. This C57BL/6 B16 melanoma cell line was generated in Peter Stern's laboratory [26]. Firstly, we confirmed h5T4 expression by flow cytometry (Figure 3A) and, next, we assessed vaccine efficacy in a tumour challenge model. For this purpose, mice were primed with ChAdOx1 viruses expressing either h5T4 or h5T4Ii, boosted twice at three week intervals with MVA and subsequently challenged subcutaneously into the right flank with B16.h5T4 cells. Once more, unvaccinated naïve mice were used as controls. Upon the appearance of palpable tumours, mice were monitored at regular intervals and tumour volumes were measured until the first animal in the group reached a pre-defined humane endpoint.

Results of a representative experiment are shown in Figure 3B–3D. B16 melanoma is known to be an aggressive tumour model and, in our hands, naïve control mice had to be sacrificed approximately two weeks post challenge due to tumour size. In contrast, the onset of tumour growth was delayed in mice vaccinated against h5T4 and h5T4Ii antigens. In fact, h5T4 vaccination resulted in complete tumour protection in five out of six mice, with only one mouse developing a tumour, as late as day 48 post challenge (Figure 3B). To assess the durability of the induced protective immune response, the remaining five tumour-free mice were re-challenged into the opposite flank approximately two months after the last immunisation. Again, tumour growth was monitored regularly and compared with a newly challenged group of naïve mice. Similarly to results obtained in the first challenge, tumour growth curves of each mouse in control and experimental groups show significant tumour growth inhibition in the vaccinated group (Figure 3C). These data demonstrate that h5T4 vaccination is not only highly efficacious in prophylactic settings, but also that immune responses against h5T4 are durable and able to prevent cancer development upon a second encounter with tumour cells.

At the end of the experiment, the immune response was further tested using total and CD8+ T cells enriched splenocytes in an ELISpot assay. Average purity of CD8+ T cells after enrichment was ~90% (data not shown). Representative images of ELISpot plate wells shown in Figure 3D indicate CD8+ T cells as responsible for the h5T4-specific IFN-γ responses elicited by vaccination, therefore suggesting that they are the main cell type involved in tumour rejection and elimination.

### Therapeutic efficacy of the h5T4-targeting vaccine in the B16.h5T4 tumour challenge model

**Figure 4:**
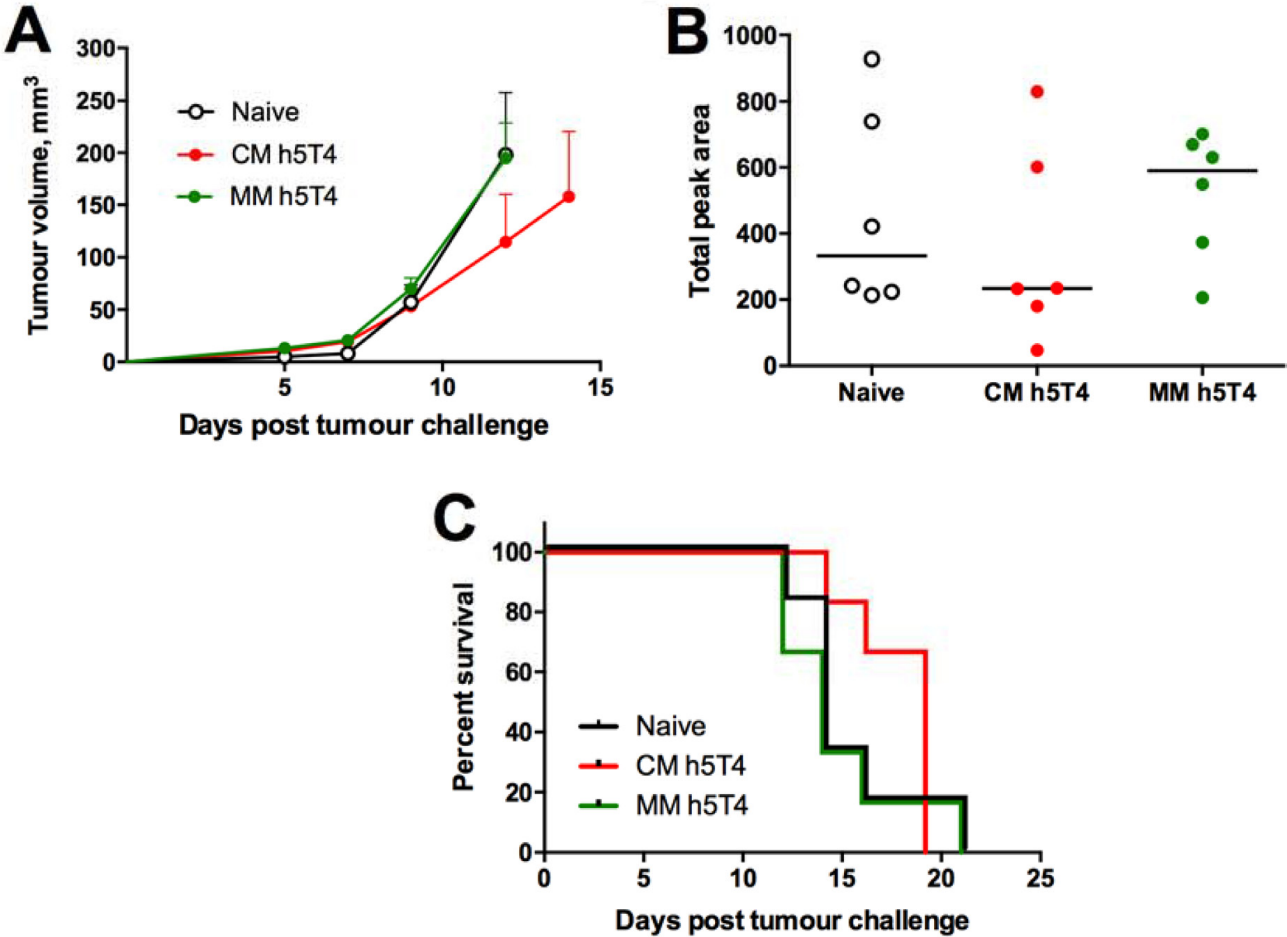
Therapeutic vaccination against h5T4 is modestly protective in B16.h5T4 melanoma tumour model C57BL/6 mice were challenged subcutaneously with 0.5*10^6^ B16.h5T4 cells and immunised intramuscularly on the same day with 10^7^ IU of ChAdOx1.h5T4 or 10^6^ pfu of MVA.h5T4. One week post challenge/prime, the two groups of mice were injected intramuscularly with 10^6^ pfu of MVA.h5T4. Control mice were challenged but left unvaccinated (naïve). Tumour size was measured three times per week and volumes were calculated as described above. (**A**) Tumour growth kinetics for each group expressed by mean tumour volume ± SEM. (**B**) assessment of vaccine efficacy by area under the curve (AUC) analysis at day 12 post B16.h5T4 cell inoculation. (**C**) Kaplan-Meyer survival curves of the three groups of mice. Bars represent median. Representative data of three biological replicate experiments are shown.

Having demonstrated a remarkable, though surprising, superiority of h5T4 over h5T4Ii vaccination in preventing tumour development, subsequent experiments to assess vaccine therapeutic efficacy were performed with vectors expressing the native h5T4 antigen. Due to aggressiveness of the B16 tumour model, it was necessary to optimise our vaccination regime for therapeutic efficacy testing. We have previously shown for the STEAP1 tumour antigen that a one log reduced dose of the vaccines given at one week intervals was able to stimulate IFN-γ responses comparable with the ones generated by the standard dose immunisations. Also, this accelerated reduced-dose regime translated into superior protection against tumour challenge in the TRAMP-C1 tumour model [17]. Therefore, we deployed this vaccination regime in a B16.h5T4 therapeutic efficacy setting. In a typical experiment, all mice were inoculated with tumour cells and test group mice were dosed with a priming vaccine on the same day due to the rapid growth of B16 cells. Of note, mice in one of the test groups were primed with MVA.h5T4 vector in order to compare ChAdOx1-MVA with the homologous MVA vaccination platform and assess the potential contribution of the h5T4-specific antibody response to tumour protection. Representative results are shown in Figure 4. While tumour growth rates were identical in naïve and MVA prime-boosted mice, the ChAdOx1-MVA vaccinated group showed a delay in tumour progression (Figure 4A). Single numerical values for individual tumour growth curves were obtained in an AUC analysis and demonstrated a trend towards efficacy of the heterologous vaccination in delaying tumour progression (Figure 4B). Also, ChAdOx1-MVA vaccination was able to slightly prolong survival of mice after tumour challenge (Figure 4C), indicating that the heterologous immunisation regime is only modestly protective against established tumours.

### Therapeutic efficacy of the h5T4-targeting vaccine in combination with immune checkpoint inhibitors (CPIs)

In order to improve therapeutic efficacy of the heterologous h5T4-targeting vaccination regime in the B16.h5T4 model, we next investigated whether we could achieve synergistic anti-tumour effects by combining the vaccine with immune checkpoint inhibitors. Blockade of immune suppressive signals using CPIs proved to be very successful as a monotherapy, having provided clinical benefit for some patients with several cancer types [27], and it has been under further intensive investigation both in clinical [28] as well as in preclinical settings [29–32]. Combating tumour adaptive immune resistance in combination with an antigen-specific vaccine seems a logical way forward towards improving clinical efficacy of cancer immunotherapies.

**Figure 5:**
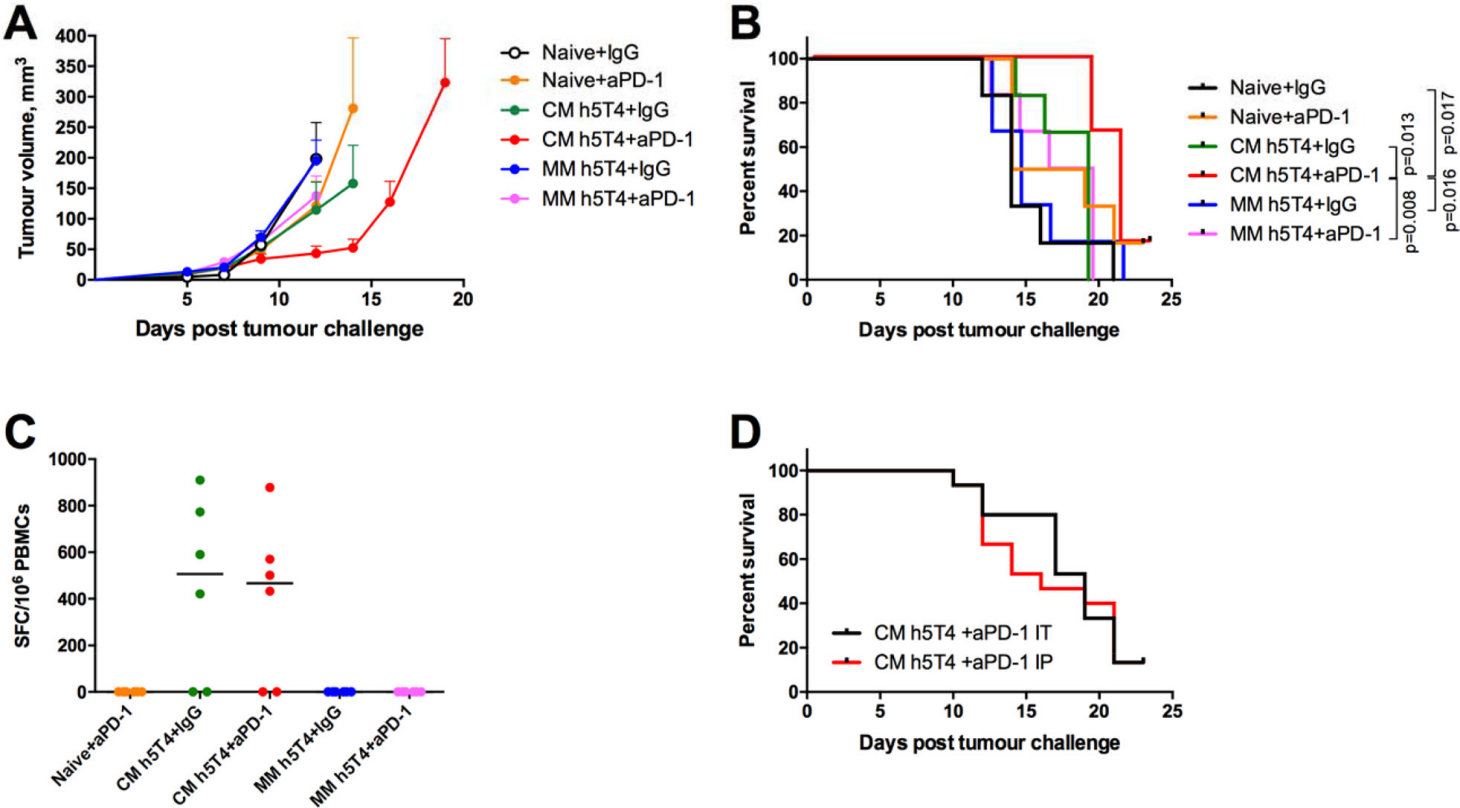
Heterologous ChAdOx1-MVA h5T4 vaccination regime in combination with anti PD-1 therapy significantly improves survival in B16.h5T4 melanoma tumour model compared with homologous MVA h5T4 vaccination combined to anti PD-1 therapy C57BL/6 mice were challenged subcutaneously with 0.5*10^6^ B16.h5T4 cells and immunised intramuscularly with 10^7^ IU of ChAdOx1.h5T4 or 10^6^ pfu of MVA.h5T4 the same day. One week later, mice were boosted with 10^6^ pfu of MVA.h5T4 with PD-1 antibody or isotype control antibody given intratumourally as described in M&M. Naïve control mice were challenged and given only antibodies (IgG or PD-1) at the timepoint for boosting immunisation. Tumour size was measured three times per week and volumes were calculated as described above. Representative data of two biological replicate experiments are shown. (**A**) Tumour growth kinetics for each group expressed by mean tumour volume ± SEM. (**B**) Kaplan-Meyer survival curves of the different groups of mice. (**C**) Graph shows representative data of *ex vivo* blood ELIspot performed after prime-boost immunisations in the different groups of mice. Bars represent median spot forming cells (SFC) per 10^6^ PBMCs. (**D**) Cumulative survival curves from two independent experiments, where mice vaccinated with ChAdOx1.h5T4-MVA.h5T4 vaccine received anti PD1 combination therapy given intraperitoneally (IP) or intratumourally (IT).

We have previously demonstrated that blockade of PD-1 receptor in combination with STEAP1 heterologous vaccination enhances efficacy and significantly improves survival of mice in the TRAMP-C1 subcutaneous tumour model [17]. Therefore, we set out to compare efficacy of the heterologous regime versus the MVA only regime with or without anti PD-1 therapy. To this end, C57BL/6 mice were challenged with B16.h5T4 cells and primed with ChAdOx1.h5T4 or MVA.h5T4 vectors, or were left untreated (naïve mice). One week after, mice received MVA.h5T4 boost and PD-1 or isotype control antibodies intraperitoneally (i.p.). A comparative efficacy of the vaccine and PD-1 blockade as monotherapies and their combinations is shown in Figure 5A, 5B. Firstly, blockade of PD-1 on its own offered just marginally better survival over isotype control mAb indicating that anti-PD-1 mAb monotherapy is not potent in this model. As expected, ChAdOx1-MVA vaccine on its own delayed tumour progression to some extent. Notably, the administration of this vaccine in combination with anti PD-1 was significantly more effective at suppressing tumour growth and prolonging survival of challenged mice than ChAdOx1-MVA vaccination used as monotherapy. In comparison, MVA-MVA dosing regime combined with either PD-1 or isotype control antibodies failed to improve survival of B16.h5T4 challenged mice, with animals succumbing to tumours at the same time as naïve controls. Importantly, the analysis of IFN-γ responses in PBMCs isolated from the different groups demonstrated no effect of anti PD-1 therapy on the magnitude of h5T4-specific T cell responses in circulation, suggesting that anti PD-1 antibodies are acting at the tumour site (Figure 5C).

Furthermore, we aimed to investigate whether local administration of PD-1 antibody at the tumour site was more efficacious than systemic delivery. If proved true, such a finding could potentially have important implications for the clinic, in that much lower doses of the drug should be required, thus diminishing the severity and incidence of adverse events in cancer patients. To this end, mice were randomised into two groups and were immunised with ChAdOx1-MVA encoding h5T4 following the experimental settings mentioned above. At the boost vaccination, one group received PD-1 mAb intraperitoneally, while mice in the second group were given the antibody intratumourally. Pooled data from two independent experiments show that the local administration of anti PD-1 therapy did not offer an overall survival advantage over the systemic delivery, although there is a trend for a slight delay in tumour growth following intratumoural administration compared with intraperitoneal PD-1 mAb injection (Figure 5D).

**Figure 6:**
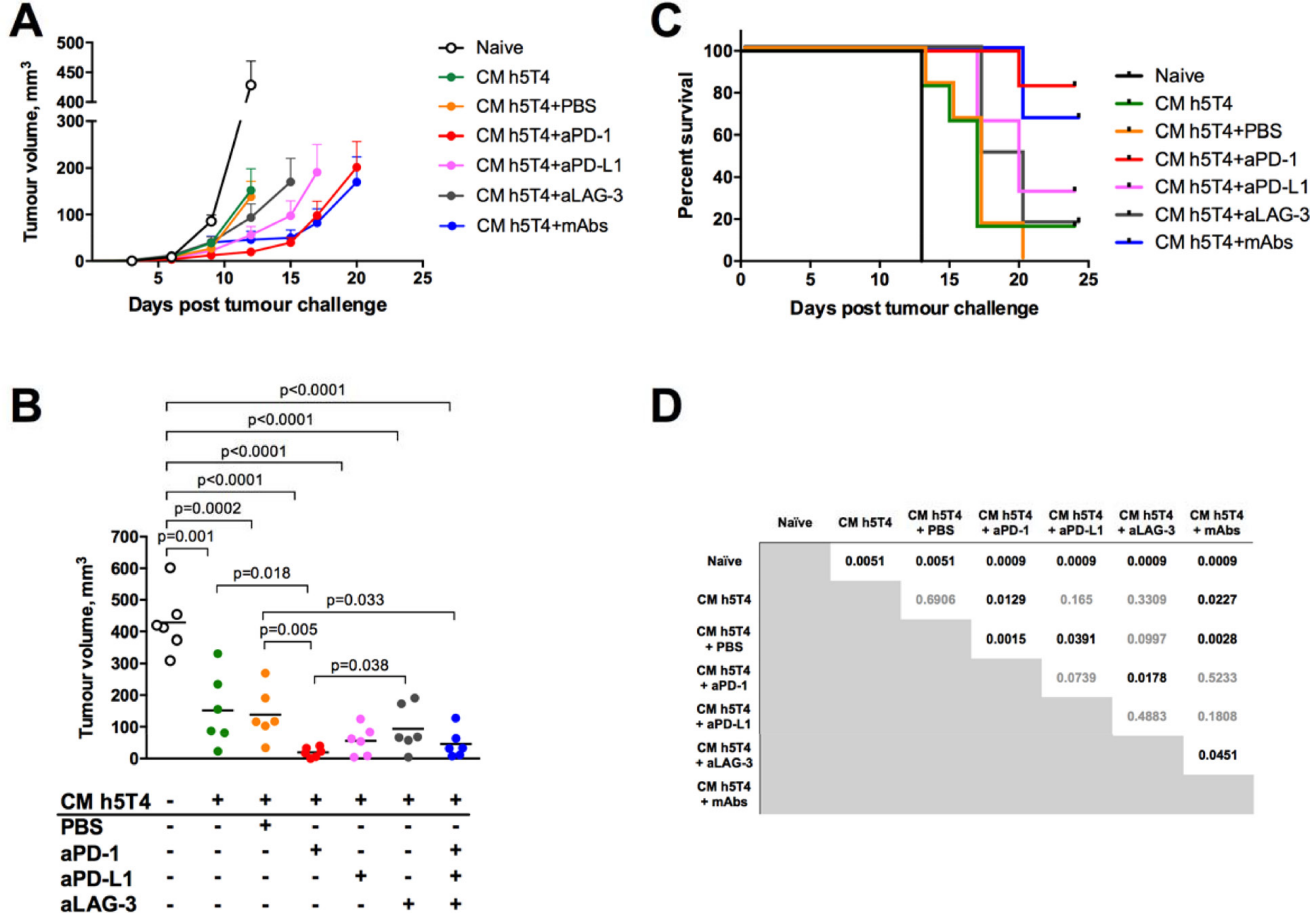
Therapeutic vaccination against h5T4 benefits significantly from the combination with PD-1 antibody compared to other checkpoint inhibitors C57BL/6 mice were challenged subcutaneously with 0.5*10^6^ B16.h5T4 cells and immunised intramuscularly with 10^7^ IU of ChAdOx1.h5T4 the same day. A week later, mice were boosted with 10^6^ pfu of MVA.h5T4 and treated intratumourally with PD-1, PD-L1, LAG-3 antibodies or a combination of all three as described in M&M. Control mice were challenged with tumour cells but were left unvaccinated (naïve). Tumour size was measured three times per week and volumes were calculated as described above. Representative data of two biological replicate experiments are shown. (**A**) Tumour growth kinetics for each group expressed by mean tumour volume ± SEM. (**B**) Tumour volumes of each individual group at day 13 post B16.h5T4 challenge. Bars represent median. (**C**) Kaplan-Meyer survival curves of the different groups of mice and (**D**) statistical analyses for each group evaluated by the log-rank assay. Significant *p* values are shown.

Finally, we extended the panel of CPIs to test PD-L1 and LAG-3 mAbs alongside anti PD-1. C57BL/6 mice were challenged with B16.h5T4 cells and randomised to receive ChAdOx1.h5T4 or no vaccine. One week later, vaccinated mice were boosted with MVA.h5T4 as a single agent, MVA.h5T4 concurrently with one of the antibodies (PD-1, PD-L1 or LAG-3), or injected with a mixture of all three mAbs. ChAdOx1-MVA monotherapy, as well as each combinatorial treatment, delayed tumour progression compared to naïve controls (Figure 6A). Tumour volumes measured at day 13 post challenge, when all mice were still alive, illustrate that only vaccines combined with anti PD-1 or a mixture of all three antibodies were able to significantly reduce tumour burden in comparison with the vaccine as a monotherapy, resulting in a dramatic inhibition of tumour growth (Figure 6B). However, we also observed a trend towards delayed tumour growth following combinations of the vaccine with anti LAG-3 and PD-L1 therapy. Overall, the survival rate of mice in each treatment group was significantly higher compared to naïve controls. Strikingly, combination of the vaccine with PD-1 antibody only, or with the mixture of all three antibodies, resulted in significant survival benefit compared with the vaccine only group, showing 80% and 65% of the animals surviving tumour challenge respectively (Figure 6C). Thus, PD-1 receptor blockade concurrently with the ChAdOx1-MVA 5T4 vaccine is the combination mediating the highest tumour protective effect, although some benefit of anti PD-L1 and LAG-3 therapy cannot be ruled out in this setting.

## DISCUSSION

Results presented in this study describe the advantage of targeting the 5T4 tumour antigen through a heterologous ChAdOx1-MVA vaccination platform over homologous MVA regime in that it induces strong tumour protective CD8+ T cell responses in preclinical settings. In addition, we have shown an enhanced efficacy of ChAdOx1-MVA based cancer vaccines when delivered in combination with anti PD-1 treatment.

It has been widely accepted that CD8+ T cells are essential for controlling tumour growth, with numerous studies demonstrating that intratumoral T-cell infiltrates, and CD8+ T cells in particular, correlate with better prognosis and clinical outcome in diverse tumour types [33–38]. Therefore, a successful cancer vaccine should be able to induce strong cellular immunity against target tumour antigens.

In the past, heterologous prime-boost vaccination regimens based on ChAdOx and MVA viruses have been shown to generate strong, durable and protective antigen-specific T cell responses in infectious disease clinical settings [11–13, 16, 39]. More recently, this vaccination platform has proved efficacious in a mouse model of prostate cancer [17]. In the current study, a single immunisation with ChAdOx1 virus expressing the human tumour-associated antigen 5T4 was sufficient to induce detectable antigen-specific T cell responses compared to the MVA.h5T4 immunisation in C57BL/6 mice. A boosting vaccination with MVA.h5T4 has increased the frequency of 5T4-specific T cells induced by ChAdOx1.h5T4 prime, whereas antigen-specific T cell responses were still undetectable following the homologous MVA boost. Of note, 5T4-specific antibody responses of comparable magnitude were induced by both ChAdOx1-MVA and MVA-MVA vaccination regimes. This observation is consistent with earlier studies describing the induction of strong h5T4-specific humoral responses, but weak [18] or undetectable [19] cellular responses following MVA vaccination. Also, a combination of MVA with avian poxvirus vectors expressing h5T4 failed to induce antigen specific CD8+ T cell responses [18]. On the contrary, the ChAdOx1-MVA regimen presented herein was a potent inducer of CD8+ T cell responses. In line with our data, Ali and colleagues pointed out the importance of the adenoviral vector Ad.h5T4 as a priming vaccine in the induction of strong antigen-specific Th1 immune responses that could be boosted by dendritic cells expressing h5T4, thus ultimately leading to therapeutic efficacy of the vaccines [40].

By linking h5T4 antigen to the MHC class II invariant chain (Ii), we were able to significantly enhance antigen-specific immune responses. Our results are consistent with several studies demonstrating that fusion with Ii improves antigen presentation and is able to accelerate, broaden and increase CD8+ T cell responses against pathogens [41–44] and tumour self-antigens following immunization with adenovirus-vectored vaccines [21, 22]. The mechanisms behind this phenomenon are yet to be elucidated, although there is an indication that Ii could be involved in the endolysosomal cross-presentation pathway mediated by dendritic cells [45].

Encouraged by the high immunogenicity of the vaccine, we moved on to efficacy studies using the TRAMP-C1 tumour cell line in challenge experiments. We have demonstrated that TRAMP-C1 cells, originally derived from the mouse adenocarcinoma of the prostate, express 5T4 along with other defined prostate-associated antigens. As mouse and human 5T4 proteins share ~80% homology [25] and we had observed some cross-reactivity of h5T4-specific T cells with the mouse antigen, we were expecting these cells to protect against TRAMP-C1 tumours to some extent. Contrary to our expectations, strong immunity against h5T4 was not sufficient to translate into retardation of tumours expressing the mouse antigen. In fact, a similar observation was reported for another tumour antigen, gp100 [46]. Murine CTLs generated by vaccination against human gp100 cross-reacted between human and mouse gp100, indicating the recognition of a conserved epitope. However, these CTLs did not appear to be involved in tumor protection.

In contrast, ChAdOx1-MVA vaccines expressing h5T4 and h5T4Ii showed a prominent prophylactic efficacy in the mouse B16 melanoma model, where tumour cells were stably transfected to express the human 5T4 protein. Importantly, all but one of the mice that had been completely protected against tumour development remained tumour-free after re-exposure to the tumour cells, pointing to strong immunological memory induced by the vaccines. Moreover, these mice manifested very high IFN-γ responses that were attributable primarily to CD8+ T cells. Similarly, Ali and colleagues showed that *in vitro* killing of h5T4-expressing tumour cells by h5T4-reactive splenocytes was completely abrogated by incubation of the splenocytes with CD8-specific antibody prior to the assay. Importantly, the heterologous vaccination used, based on adenovirus and DC cells, was only effective in therapeutic settings when Ad was used as a priming agent [40]. Thus, we can conclude that our ChAdOx1-MVA prime boost regime is a potent inducer of durable antigen-specific CD8+ T cell responses, which are ultimately responsible for tumour rejection. The 5T4-specific antibody response that has been identified by others as a major player in tumour elimination [18, 47], could be ruled out as a mediator of protection in our study because of two findings. Firstly, ChAdOx1-MVA expressing 5T4 in its native and li-linked forms elicited comparable antigen-specific antibody response, but controlled tumour growth differently in prophylactic settings. Secondly, the homologous MVA.h5T4 immunisation which induced comparable antibody responses to the heterologous prime-boost regime completely failed to protect mice from the growth of established B16.h5T4 tumours.

Surprisingly, the higher frequency of IFN-γ-secreting T cells following h5T4Ii vaccination did not translate into a better efficacy in the B16 melanoma model, when vaccines were given as prophylactic treatment. We had previously observed a similar effect in the TRAMP-C1 tumour model using the same vectored vaccines expressing a different prostate cancer associated antigen in its native or Ii-fused forms (unpublished data). Our results are in contrast with the data published by Sorensen and colleagues showing superiority of the Ii fusion with LCMV glycoprotein as the vaccine target antigen, delivered by Ad5 in a B16 melanoma model expressing an immunodominant peptide from LCMV glycoprotein as a tumour neoantigen [21]. The reasons for the discrepancy between our data and the LCMV results are unclear. A possible explanation could be that stronger T cell responses result in stronger adaptive immune resistance mounted by tumours in response to the immune attack in line with the concept of the cancer-immunity cycle [48]. Indeed, it has been shown that immunization of tumour-bearing mice with an optimized more immunogenic vaccine encoding altered epitopes with increased MHC class I affinity elicited a surprisingly inferior anti-tumour effect alongside higher expression of PD1 on the antigen-specific T cells relative to the native vaccine [32]. Possibly, in our model also the higher activation status of antigen-specific T cells following the 5T4.Ii immunization resulted in more profound T cell exhaustion and, therefore, inferior anti-tumour protection.

Despite its high efficacy as a preventive cancer vaccine, ChAdOx1-MVA vaccination against h5T4 only modestly delayed tumour growth and marginally improved survival of B16.h5T4 tumour-bearing mice. In agreement with our data, MVA.h5T4 homologous vaccination has been previously shown to protect from B16 melanoma tumours, but has only marginally increased survival of mice with established tumours [47]. These results support once more the view that the active adaptive immunosuppressive processes that tumours rapidly develop following vaccination, are a major factor limiting tumour regression. For instance, in a murine melanoma model, tumour-bearing mice were only modestly protected despite a robust antigen-specific CD8+ T cell response following vaccination with a potent recombinant adenovirus [49]. In the same study, tumour-infiltrating lymphocytes were found to be less functional compared to PBMCs and, moreover, they were found to progressively lose their function after vaccination. The authors showed that immunosuppression directly correlated with the magnitude of the immune response, and that it was mainly the IFN-γ released by CD8+ T cells early after tumour infiltration that was responsible for the rapid induction of immunosuppressive pathways in the tumour. This is in line with other studies reporting that IFN-γ secreted by CD8+ T cell tumour infiltrates are responsible for at least three suppressive mechanisms, i.e. the up-regulation of both PD-L1 and IDO on tumour cells and recruitment of regulatory T cells into the tumour microenvironment [50]. In addition, the tumour microenvironment itself has the ability to convert infiltrated CD8+ effector cells into suppressor cells, thus contributing to the partial inefficiency of the immune response in controlling tumour growth following immunotherapy [51]. Again, we can speculate that the pronounced immunogenicity of h5T4 and, in particular, h5T4Ii vaccinations could enhance such mechanisms of immune escape, thus reducing vaccine efficacy against established tumours. Further investigation is also needed to analyse whether the cytotoxicity and functional avidity of CD8+ T cells are differently regulated following heterologous versus homologous vaccination or heterologous vaccination against the native 5T4 or the Ii-linked antigen, as it has been shown that high-avidity T cells are preferentially tolerized in the tumour microenvironment [52].

To improve the ChAdOx1-MVA h5T4 vaccine efficacy in therapeutic settings, we set out to evaluate a combination immunotherapy with monoclonal antibodies against checkpoint targets to counteract the mechanisms blunting the induced immune responses. PD-1 up-regulation on activated T cells in circulation and in the tumour microenvironment has been well documented, as has the upregulation of its ligand, PD-L1, by tumour cells and several immune cell subsets (reviewed by Ostrand-Rosenberg [53]). More efficient tumour rejection has been observed when peptide- and cell-based vaccines were combined with blockade of one or more checkpoint receptors [29, 30]. Recently, we have provided evidence that PD-1 blockade in combination with a ChAdOx1-MVA immunisation regime against STEAP1 antigen is successful in improving survival and reducing tumour burden in a transplantable mouse model of prostate cancer [17]. In the present study, ChAdOx1-MVA vaccination did significantly benefit from the addition of anti PD-1 therapy. In contrast, neither the MVA homologous regime in combination with PD-1 antibody, nor anti PD-1 therapy alone, were able to protect against tumour outgrowth and improve survival of tumour-bearing mice. Our results are consistent with other studies that have used anti PD-1 therapy in combination with the cell-based TLR agonist enhanced vaccine TEGVAX in the B16 melanoma model and with a DNA vaccine in a murine sarcoma tumour model [31, 32]. The fact that h5T4-specific T cells responses in blood were of a similar magnitude following treatment with the vaccine as a single agent and in combination with PD-1 antibody, might indicate that anti PD-1 therapy positively regulates immune responses within the tumour microenvironment.

Aiming at the translation of our experimental work into the clinic, we attempted to optimise the combination therapy by delivering checkpoint blockers intratumourally at a reduced dose, in order to minimize potential immune-related adverse effects attributable to the systemic administration. After an initial slight delay of the tumour growth in mice given anti PD-1 intratumorally, eventually the survival rates evened out between the mice given the antibody intratumourally and intraperitoneally. However, we believe that intratumoural administration of CPIs can be more efficacious than systemic delivery in other less aggressive mouse tumour models, and we will continue to evaluate this possibility. If proved correct in mouse models, this route of administration should be considered for clinical testing in suitable cancer types.

Although we could significantly improve survival of mice using the combination treatment, tumour protection was not complete. Despite the success of immune checkpoint blockade across several cancer types in clinic [54], resistance to treatment and relapse are common. As many tumour cells express multiple inhibitory ligands and TILs express multiple inhibitory receptors, dual or triple blockade of immune checkpoints may enhance antitumour immunity. For example, in the context of tumours, exhausted T cells express a whole set of inhibitory receptors with likely irredundant functions, such as CTLA4, TIM3, LAG3, TIGIT and others [55]. The number of currently ongoing clinical trials testing various combinations of immunomodulatory agents across cancer types is well over 1500.

In the present study, we have chosen to test a triple blockade of PD-1, PD-L1 and LAG-3 alongside vaccination. In our hands, the combination of ChAdOx1-MVA regime with the blockade of PD-1 alone had the same effect as the triple blockade of PD-1, PD-L1 and LAG-3 in terms of growth inhibition and survival. A previous study has shown that anti LAG-3/anti PD-1 combinatorial immunotherapy was not effective against established B16 melanoma tumours, as opposed to fibrosarcoma and colorectal adenocarcinoma tumour models [56]. In comparison, targeting PD-1/PD-L1 pathway and LAG-3 has been shown to synergistically increase the frequency and restore effector functions of tumour-specific CD8+ T cells in human ovarian cancer [57]. More recently, Foy and colleagues observed complete regression of CT26.HER-2 tumours combining immunotherapy with dual PD-1 and LAG-3 blockade [58]. Based on such variable outcomes of combination therapies across mouse tumour models and clinical trials, we can speculate that very diverse, often redundant, mechanisms are likely to operate in different models and species. We believe that the therapeutic benefit observed by combining ChAdOx1-MVA heterologous vaccination and PD-1 blockade could be improved by the characterization of the unique immune regulatory pressure in the B16 melanoma model, and so we are addressing additional immune escape mechanisms in our current experiments.

In conclusion, preclinical data presented herein demonstrate the high translational potential of the vaccination platform based on ChAdOx1 and MVA viral vectors expressing h5T4 in combination with PD-1 blockade for cancer immunotherapy and provide a foundation for further clinical development.

## MATERIALS AND METHODS

### Mice and cell lines

6 week old male C57BL/6 mice used in this study were purchased from Harlan, UK. Mouse care and experimental procedures were carried out in accordance with the terms of the UK Animals (Scientific Procedures) Act Project License (PPL 30/2947) and approved by the University of Oxford Animal Care and Ethical Review Committee.

TRAMP-C1 cell line was purchased from the American Type Culture Collection (ATCC) and maintained in high glucose DMEM supplemented with 5% fetal bovine serum, 5% NuSerum (Becton Dickinson), 4 mM L-Gln, 1% Pen-Strep, 10 nmol/L dihydrotestosterone (Sigma), and 5 μg/mL bovine insulin (Sigma).

B16.h5T4 cell line was a kind gift from Peter Stern (Paterson Institute for Cancer Research, Manchester, UK) and maintained in DMEM supplemented with 10% fetal bovine serum, 4 mM L-Gln and 1 mg/ml G418.

### ChAdOx1 and MVA viral vector construction

The DNA sequences encoding human 5T4 antigen (h5T4) (NCBI RefSeq NM_001166392.1), its mouse counterpart m5T4 (NCBI RefSeq NM_011627.4) and a fusion construct of h5T4 to full length human invariant chain (Ii) (NCBI RefSeq NP_004346.1) were obtained from GeneArt (Life technologies, Paisley, UK). The h5T4.Ii construct was designed as an in-frame fusion of the N terminus of the h5T4 cDNA sequence, with the signal peptide deleted to the C terminus of the full length human invariant chain. The construction of ChAdOx1 vectors was described earlier [59]. Briefly, the full length h5T4 and m5T4 cDNA and h5T4Ii fusion under a CMV immediate early promoter were sub-cloned from a pENTR plasmid into the E1 locus of the pBAC ChAdOx1-DEST genomic clone by *in vitro* site-specific recombination (GatewayTM cloning). The constructs were then used to transfect HEK293A cells to generate the recombinant adenoviruses expressing the antigens. The MVA.GFP shuttle vector drives the expression of h5T4 and m5T4 under the P7.5 early/late promoter inserted at the thymidine kinase locus of MVA and the GFP from the fowlpox FP4b late promoter. The plasmids were transfected into MVA-infected primary chick embryo fibroblasts (CEFs; Institute for Animal Health, Compton, UK) and recombinant viruses were isolated by selection of GFP-positive plaques, amplified, purified over sucrose cushions and titred in CEFs according to standard practice. The integrity, identity and purity of the viruses were confirmed by PCR analysis.

### *In vivo* studies: immunogenicity and tumour models

Human and mouse native 5T4 antigen and h5T4Ii fusion were tested in immunogenicity and efficacy studies as follows. A dose of 10^8^ infectious units (IU) of ChAdOx1 or 10^7^ plaque forming units (PFU) of MVA expressing m5T4, h5T4, or h5T4Ii were diluted in DPBS and injected intramuscularly (50 μl per animal) in C57BL/6 mice. In alternative experiments, virus doses were reduced by one log. Immunisations were performed weekly or alternatively at 3–4 weeks intervals, and immune responses in blood or spleen were measured at indicated time points. Total number of vaccinations ranged between 2 and 4.

To assess vaccine tumour-protective efficacy in a transplantable tumour model, 2 × 10^6^ TRAMP-C1 or 0.5 × 10^6^ B16.h5T4 cells were injected subcutaneously (s.c.) into the right flank of C57BL/6 male mice in a total volume of 100 μl DPBS. Tumour growth was monitored 3 times weekly and mice were sacrificed when tumour size reached 10mm in any direction. Tumour volume was calculated by the following formula: length (mm) × width^2^ (mm) × 0.52.

In combination studies, the monoclonal antibodies against mouse PD-1 (clone RMP1-14), PD-L1 (clone 10F.9G2), and LAG3 (clone C9B7W) or Rat IgG2a isotype control antibody (clone 2A3) were administered intraperitoneally (i.p.) or intratumourally (i.t.) at the dose of 50 μg per mouse at indicated time points.

### Measurement of 5T4-specific immune responses by IFN-γ Elispot

An *ex vivo* IFN-γ Elispot assay was performed using Multiscreen IP ELISPOT plates (Millipore) and mIFN-γ Elispot kit (ALP) (Mabtech). Mouse and human 5T4 peptide libraries were synthesized by Mimotopes (UK) and consisted of 15-mer peptides overlapping by 10 amino acids spanning the whole protein length. Individual peptide pools or a mix of all pools at a final concentration of 5 μg/ml were used to stimulate PBMCs or splenocytes for 18–22 hours prior to detection of spot-forming cells (SFCs). Enrichment of splenocytes for CD8 positive T cells was performed by negative selection using a mouse CD8a^+^ T Cell Isolation Kit (Miltenyi Biotec). ELISpot plates were read using an AID automated ELISPOT counter (AID Diagnostika GmbH) using identical settings for all plates.

### Measurement of 5T4-specific immune responses by ELISA

Anti-5T4 antibody responses were measured by enzyme-linked immunosorbent assay (ELISA) against recombinant 5T4 protein at 2.5 ug/ml (kind gift from Richard Harrop, OxfordBiomedica Ltd.) as described earlier [18]. Endpoint titres of 5T4-specific antibodies in vaccinated mice were calculated as the highest serum dilution that yielded at least a 2 fold greater OD compared to the naïve serum.

### Flow cytometry: 5T4-specific immune responses and expression

Mouse PBMCs or splenocytes were stimulated *ex vivo* with 5T4 peptide pools (5 μg/ml of each peptide) and 1 μg/ml Golgi-Plug (BD) for 6 hours. Following stimulation, Fc receptors were blocked with anti mouse CD16/32 and cells were labelled with anti-mouse CD4-AlexaFluor700, and CD8-PerCPCy5.5 (eBioscience Ltd), fixed-permeabilized in CytofixCytoperm buffer (BD) and incubated with IFN-γ-PE, IL-2-APC and TNF-α-FITC antibodies (eBioscience Ltd).

To confirm 5T4 expression on the surface of the B16.h5T4 cell line, cells were gently dissociated and incubated with anti 5T4 antibody (clone H8, kind gift from Richard Harrop, Oxford BioMedica Ltd.) or mouse IgG isotype control antibody. Anti mouse IgG-FITC was used as secondary antibody.

All sample acquisitions were performed on a BD LSRII™ analyzer and data analysed with FlowJo software (Treestar).

### Analysis of 5T4 gene expression

5T4 mRNA expression in TRAMP-C1 cells and thymi of C57BL/6 mice was detected by semi-quantitative reverse transcription PCR. Total RNA was extracted from cells and tissue samples with RNeasy Plus minikit (Qiagen) and a total of 1–2 μg of RNA was used to synthesize the first single-strand cDNA using QuantiTect Reverse Transcription kit (Qiagen) according to the manufacturers’ guidelines. The primers for RT-PCR amplification have been reported earlier [17].

### Statistical analysis

Data presented in this manuscript are representative of at least 2 independent experiments using a minimum of 6 animals per group. Unless stated otherwise, median values ± SEM are shown. Comparisons of ELISpot and ICS results among groups were analysed using unpaired Student's t test. Area under curve (AUC) analysis was performed to compare tumour growth kinetics in naïve versus 5T4-vaccinated mice. For comparisons between groups, a one-way ANOVA with Tukey's post-test comparison was performed. Statistical significance in survival experiments was evaluated by the log-rank test. Differences were considered statistically significant for two-sided *p* values < 0.05. Statistical analysis was performed using the GraphPad PRISM software (v6).

## References

[1] Southall PJ, Boxer GM, Bagshawe KD, Hole N, Bromley M, Stern PL (1990). Immunohistological distribution of 5T4 antigen in normal and malignant tissues. British Journal of Cancer.

[2] Starzynska T, Marsh PJ, Schofield PF, Roberts SA, Myers KA, Stern PL (1994). Prognostic significance of 5T4 oncofetal antigen expression in colorectal carcinoma. Br J Cancer.

[3] Amato RJ, Stepankiw M (2012). Evaluation of MVA-5T4 as a novel immunotherapeutic vaccine in colorectal, renal and prostate cancer. Future Oncol.

[4] Stern PL, Brazzatti J, Sawan S, McGinn OJ (2014). Understanding and exploiting 5T4 oncofoetal glycoprotein expression. Seminars in Cancer Biology.

[5] Stern PL, Harrop R (2016). 5T4 oncofoetal antigen: an attractive target for immune intervention in cancer. Cancer Immunology, Immunotherapy.

[6] Harrop R, Connolly N, Redchenko I, Valle J, Saunders M, Ryan MG, Myers KA, Drury N, Kingsman SM, Hawkins RE, Carroll MW (2006). Vaccination of Colorectal Cancer Patients with Modified Vaccinia Ankara Delivering the Tumor Antigen 5T4 (TroVax) Induces Immune Responses which Correlate with Disease Control: A Phase I/II Trial. Clinical Cancer Research.

[7] Kim DW, Krishnamurthy V, Bines SD, Kaufman HL (2010). Trovax, a recombinant modified vaccinia ankara virus encoding 5T4. Lessons learned and future development. Human Vaccines.

[8] Al-Taei S, Salimu J, Lester JF, Linnane S, Goonewardena M, Harrop R, Mason MD, Tabi Z (2012). Overexpression and potential targeting of the oncofoetal antigen 5T4 in malignant pleural mesothelioma. Lung Cancer.

[9] Harrop R, Shingler W, Kelleher M, de Belin J, Treasure P (2010). Cross-trial analysis of immunologic and clinical data resulting from phase I and II trials of MVA-5T4 (TroVax) in colorectal, renal, and prostate cancer patients. Journal of immunotherapy.

[10] Harrop R, Treasure P, Belin J, Kelleher M, Bolton G, Naylor S, Shingler WH (2012). Analysis of pre-treatment markers predictive of treatment benefit for the therapeutic cancer vaccine MVA-5T4 (TroVax). Cancer Immunology, Immunotherapy.

[11] Ewer KJ, O’Hara GA, Duncan CJ, Collins KA, Sheehy SH, Reyes-Sandoval A, Goodman AL, Edwards NJ, Elias SC, Halstead FD, Longley RJ, Rowland R, Poulton ID (2013). Protective CD8+ T-cell immunity to human malaria induced by chimpanzee adenovirus-MVA immunisation. Nat Commun.

[12] Antrobus RD, Coughlan L, Berthoud TK, Dicks MD, Hill AV, Lambe T, Gilbert SC (2014). Clinical assessment of a novel recombinant simian adenovirus ChAdOx1 as a vectored vaccine expressing conserved Influenza A antigens. Molecular therapy.

[13] Borthwick N, Ahmed T, Ondondo B, Hayes P, Rose A, Ebrahimsa U, Hayton EJ, Black A, Bridgeman A, Rosario M, Hill AV, Berrie E, Moyle S (2014). Vaccine-elicited human T cells recognizing conserved protein regions inhibit HIV-1. Molecular therapy.

[14] Swadling L, Capone S, Antrobus RD, Brown A, Richardson R, Newell EW, Halliday J, Kelly C, Bowen D, Fergusson J, Kurioka A, Ammendola V, Del Sorbo M (2014). A human vaccine strategy based on chimpanzee adenoviral and MVA vectors that primes, boosts, and sustains functional HCV-specific T cell memory. Science translational medicine.

[15] Hodgson SH, Ewer KJ, Bliss CM, Edwards NJ, Rampling T, Anagnostou NA, de Barra E, Havelock T, Bowyer G, Poulton ID, de Cassan S, Longley R, Illingworth JJ (2015). Evaluation of the efficacy of ChAd63-MVA vectored vaccines expressing circumsporozoite protein and ME-TRAP against controlled human malaria infection in malaria-naive individuals. The Journal of infectious diseases.

[16] Ewer K, Rampling T, Venkatraman N, Bowyer G, Wright D, Lambe T, Imoukhuede EB, Payne R, Fehling SK, Strecker T, Biedenkopf N, Krahling V, Tully CM (2016). A Monovalent Chimpanzee Adenovirus Ebola Vaccine Boosted with MVA. The New England journal of medicine.

[17] Cappuccini F, Stribbling S, Pollock E, Hill AV, Redchenko I (2016). Immunogenicity and efficacy of the novel cancer vaccine based on simian adenovirus and MVA vectors alone and in combination with PD-1 mAb in a mouse model of prostate cancer. Cancer immunology, immunotherapy.

[18] Harrop R, Ryan MG, Myers KA, Redchenko I, Kingsman SM, Carroll MW (2005). Active treatment of murine tumors with a highly attenuated vaccinia virus expressing the tumor associated antigen 5T4 (TroVax) is CD4+ T cell dependent and antibody mediated. Cancer Immunology, Immunotherapy.

[19] Hanwell DG, McNeil B, Visan L, Rodrigues L, Dunn P, Shewen PE, Macallum GE, Turner PV, Vogel TU (2013). Murine Responses to Recombinant MVA Versus ALVAC Vaccines Against Tumor-associated Antigens, gp100 and 5T4. Journal of immunotherapy.

[20] Alme AK, Karir BS, Faltas BM, Drake CG (2016). Blocking immune checkpoints in prostate, kidney, and urothelial cancer: An overview. Urologic oncology.

[21] Sorensen MR, Holst PJ, Pircher H, Christensen JP, Thomsen AR (2009). Vaccination with an adenoviral vector encoding the tumor antigen directly linked to invariant chain induces potent CD4(+) T-cell-independent CD8(+) T-cell-mediated tumor control. European journal of immunology.

[22] Pedersen SR, Sørensen MR, Buus S, Christensen JP, Thomsen AR (2013). Comparison of vaccine-induced effector CD8 T cell responses directed against self- and non-self-tumor antigens: implications for cancer immunotherapy. J Immunol.

[23] Krupa M, Canamero M, Gomez CE, Najera JL, Gil J, Esteban M (2011). Immunization with recombinant DNA and modified vaccinia virus Ankara (MVA) vectors delivering PSCA and STEAP1 antigens inhibits prostate cancer progression. Vaccine.

[24] Spies E, Reichardt W, Alvarez G, Groettrup M, Ohlschlager P (2012). An artificial PAP gene breaks self-tolerance and promotes tumor regression in the TRAMP model for prostate carcinoma. Molecular therapy.

[25] King KW, Sheppard FC, Westwater C, Stern PL, Myers KA (1999). Organisation of the mouse and human 5T4 oncofoetal leucine-rich glycoprotein genes and expression in foetal and adult murine tissues. Biochim Biophys Acta.

[26] Woods AM, Wang WW, Shaw DM, Ward CM, Carroll MW, Rees BR, Stern PL (2002). Characterization of the murine 5T4 oncofoetal antigen: a target for immunotherapy in cancer. Biochem J.

[27] Topalian SL, Hodi FS, Brahmer JR, Gettinger SN, Smith DC, McDermott DF, Powderly JD, Carvajal RD, Sosman JA, Atkins MB, Leming PD, Spigel DR, Antonia SJ (2012). Safety, activity, and immune correlates of anti-PD-1 antibody in cancer. The New England journal of medicine.

[28] Topalian SL, Taube JM, Anders RA, Pardoll DM (2016). Mechanism-driven biomarkers to guide immune checkpoint blockade in cancer therapy. Nature Reviews Cancer.

[29] Duraiswamy J, Kaluza KM, Freeman GJ, Coukos G (2013). Dual Blockade of PD-1 and CTLA-4 Combined with Tumor Vaccine Effectively Restores T-Cell Rejection Function in Tumors. Cancer research.

[30] Karyampudi L, Lamichhane P, Scheid AD, Kalli KR, Shreeder B, Krempski JW, Behrens MD, Knutson KL (2014). Accumulation of memory precursor CD8 T cells in regressing tumors following combination therapy with vaccine and anti-PD-1 antibody. Cancer research.

[31] Fu J, Malm I, Kadayakkara H, Levitsky H, Pardoll DM, Kim YJ (2014). Preclinical Evidence That PD1 Blockade Cooperates with Cancer Vaccine TEGVAX to Elicit Regression of Established Tumors. Cancer research.

[32] Rekoske B, Smith H, Olson B, Maricque B, McNeel D (2015). PD-1 or PD-L1 Blockade Restores Anti-Tumor Efficacy Following SSX2 Epitope-Modified DNA Vaccine Immunization. Cancer Immunology Research.

[33] Naito Y, Saito K, Shiiba K, Ohuchi A, Saigenji K, Nagura H, Ohtani H (1998). CD8+ T cells infiltrated within cancer cell nests as a prognostic factor in human colorectal cancer. Cancer research.

[34] Nakano O, Sato M, Naito Y, Suzuki K, Orikasa S, Aizawa M, Suzuki Y, Shintaku I, Nagura H, Ohtani H (2001). Proliferative activity of intratumoral CD8(+) T-lymphocytes as a prognostic factor in human renal cell carcinoma: clinicopathologic demonstration of antitumor immunity. Cancer research.

[35] Zhang L, Conejo-Garcia JR, Katsaros D, Gimotty PA, Massobrio M, Regnani G, Makrigiannakis A, Gray H, Schlienger K, Liebman MN, Rubin SC, Coukos G (2003). Intratumoral T cells, recurrence, and survival in epithelial ovarian cancer. The New England journal of medicine.

[36] Kärjä V, Aaltomaa S, Lipponen P, Isotalo T, Talja M, Mokka R (2005). Tumour-infiltrating lymphocytes: A prognostic factor of PSA-free survival in patients with local prostate carcinoma treated by radical prostatectomy. Anticancer research.

[37] Noble F, Mellows T, Matthews LH, Bateman AC, Harris S, Underwood TJ, Byrne JP, Bailey IS, Sharland DM, Kelly JJ, Primrose JN, Sahota SS, Bateman AR (2016). Tumour infiltrating lymphocytes correlate with improved survival in patients with oesophageal adenocarcinoma. Cancer Immunology, Immunotherapy.

[38] Gabrielson A, Wu Y, Wang H, Jiang J, Kallakury B, Gatalica Z, Reddy S, Kleiner D, Fishbein T, Johnson L, Island E, Satoskar R, Banovac F (2016). Intratumoral CD3 and CD8 T-cell Densities Associated with Relapse-Free Survival in HCC. Cancer Immunology Research.

[39] Sheehy SH, Duncan CJ, Elias SC, Biswas S, Collins KA, O’Hara GA, Halstead FD, Ewer KJ, Mahungu T, Spencer AJ, Miura K, Poulton ID, Dicks MD (2012). Phase Ia clinical evaluation of the safety and immunogenicity of the Plasmodium falciparum blood-stage antigen AMA1 in ChAd63 and MVA vaccine vectors. PloS one.

[40] Ali S, Mulryan K, Taher T, Stern PL (2007). Immunotherapy success in prophylaxis cannot predict therapy: prime-boost vaccination against the 5T4 oncofoetal antigen. Cancer immunology, immunotherapy.

[41] Holst PJ, Sorensen MR, Mandrup Jensen CM, Orskov C, Thomsen AR, Christensen JP (2008). MHC class II-associated invariant chain linkage of antigen dramatically improves cell-mediated immunity induced by adenovirus vaccines. Journal of immunology.

[42] Mikkelsen M, Holst PJ, Bukh J, Thomsen AR, Christensen JP (2011). Enhanced and sustained CD8+ T cell responses with an adenoviral vector-based hepatitis C virus vaccine encoding NS3 linked to the MHC class II chaperone protein invariant chain. Journal of immunology.

[43] Capone S, Naddeo M, D’Alise A, Abbate A, Grazioli F, Gaudio A, Sorbo M, Esposito M, Ammendola V, Perretta G, Taglioni A, Colloca S, Nicosia A (2014). Fusion of HCV nonstructural antigen to MHC class II-associated invariant chain enhances T-cell responses induced by vectored vaccines in nonhuman primates. Molecular therapy.

[44] Spencer AJ, Cottingham MG, Jenks JA, Longley RJ, Capone S, Colloca S, Folgori A, Cortese R, Nicosia A, Bregu M, Hill AVS (2014). Enhanced Vaccine-Induced CD8+ T Cell Responses to Malaria Antigen ME-TRAP by Fusion to MHC Class II Invariant Chain. PloS one.

[45] Basha G, Omilusik K, Chavez-Steenbock A, Reinicke AT, Lack N, Choi KB, Jefferies WA (2012). A CD74-dependent MHC class I endolysosomal cross-presentation pathway. Nature immunology.

[46] Schreurs MW, de Boer AJ, Figdor CG, Adema GJ (1998). Genetic vaccination against the melanocyte lineage-specific antigen gp100 induces cytotoxic T lymphocyte-mediated tumor protection. Cancer research.

[47] Mulryan K, Ryan MG, Myers KA, Shaw D, Wang W, Kingsman SM, Stern PL, Carroll MW (2002). Attenuated Recombinant Vaccinia Virus Expressing Oncofetal Antigen (Tumor-associated Antigen) 5T4 Induces Active Therapy of Established Tumors. Mol Cancer Ther.

[48] Chen DS, Mellman I (2013). Oncology meets immunology: the cancer-immunity cycle. Immunity.

[49] McGray A, Robin H, Dannie B, Stephanie S, Ziqian Z, Florentina T, Heather V, John AH, Arthur AH, Yonghong W, Jonathan LB (2013). Immunotherapy-induced CD8+ T cells instigate immune suppression in the tumor. Molecular Therapy.

[50] Spranger S, Spaapen RM, Zha Y, Williams J, Meng Y, Ha TT, Gajewski TF (2013). Up-regulation of PD-L1, IDO, and T(regs) in the melanoma tumor microenvironment is driven by CD8(+) T cells. Science translational medicine.

[51] Shafer-Weaver KA, Anderson MJ, Stagliano K, Malyguine A, Greenberg NM, Hurwitz AA (2009). Cutting Edge: Tumor-specific CD8+ T cells infiltrating prostatic tumors are induced to become suppressor cells. J Immunol.

[52] Zhu Z, Singh V, Watkins SK, Bronte V, Shoe JL, Feigenbaum L, Hurwitz AA (2013). High-avidity T cells are preferentially tolerized in the tumor microenvironment. Cancer research.

[53] Ostrand-Rosenberg S, Horn L, Haile S (2014). The programmed death-1 immune-suppressive pathway: barrier to antitumor immunity. J Immunol.

[54] Topalian SL, Drake CG, Pardoll DM (2015). Immune Checkpoint Blockade: A Common Denominator Approach to Cancer Therapy. Cancer Cell.

[55] Benci JL, Xu B, Qiu Y, Wu TJ, Dada H, Twyman-Saint Victor C, Cucolo L, Lee D, Pauken KE, Huang AC, Gangadhar TC, Amaravadi RK, Schuchter LM (2016). Tumor Interferon Signaling Regulates a Multigenic Resistance Program to Immune Checkpoint Blockade. Cell.

[56] Woo SR, Turnis ME, Goldberg MV, Bankoti J, Selby M, Nirschl CJ, Bettini ML, Gravano DM, Vogel P, Liu CL, Tangsombatvisit S, Grosso JF, Netto G (2012). Immune inhibitory molecules LAG-3 and PD-1 synergistically regulate T-cell function to promote tumoral immune escape. Cancer research.

[57] Matsuzaki J, Gnjatic S, Mhawech-Fauceglia P, Beck A, Miller A, Tsuji T, Eppolito C, Qian F, Lele S, Shrikant P, Old LJ, Odunsi K (2010). Tumor-infiltrating NY-ESO-1-specific CD8+ T cells are negatively regulated by LAG-3 and PD-1 in human ovarian cancer. Proc Natl Acad Sci USA.

[58] Foy SP, Sennino B, dela Cruz T, Cote JJ, Gordon EJ, Kemp F, Xavier V, Franzusoff A, Rountree RB, Mandl SJ (2016). Poxvirus-Based Active Immunotherapy with PD-1 and LAG-3 Dual Immune Checkpoint Inhibition Overcomes Compensatory Immune Regulation, Yielding Complete Tumor Regression in Mice. PloS one.

[59] Dicks MD, Spencer AJ, Edwards NJ, Wadell G, Bojang K, Gilbert SC, Hill AV, Cottingham MG (2012). A novel chimpanzee adenovirus vector with low human seroprevalence: improved systems for vector derivation and comparative immunogenicity. PloS one.

